# Human Milk Lactose, Insulin, and Glucose Relative to Infant Body Composition during Exclusive Breastfeeding

**DOI:** 10.3390/nu13113724

**Published:** 2021-10-22

**Authors:** Ali S. Cheema, Lisa F. Stinson, Alethea Rea, Ching Tat Lai, Matthew S. Payne, Kevin Murray, Donna T. Geddes, Zoya Gridneva

**Affiliations:** 1 School of Molecular Sciences, The University of Western Australia, Crawley, WA 6009, Australia; alisadiq.cheema@research.uwa.edu.au (A.S.C.); lisa.stinson@uwa.edu.au (L.F.S.); ching-tat.lai@uwa.edu.au (C.T.L.); donna.geddes@uwa.edu.au (D.T.G.); 2 Mathematics and Statistics, Murdoch University, Murdoch, WA 6150, Australia; alethea.rea@murdoch.edu.au; 3 Division of Obstetrics and Gynaecology, UWA Medical School, The University of Western Australia, Crawley, WA 6009, Australia; matthew.payne@uwa.edu.au; 4 Women and Infants Research Foundation, Subiaco, WA 6008, Australia; 5 School of Population and Global Health, The University of Western Australia, Crawley, WA 6009, Australia; kevin.murray@uwa.edu.au

**Keywords:** lactose, glucose, insulin, human milk, breastfeeding, infant, body composition, bioelectrical impedance spectroscopy, intake, lactation

## Abstract

Human milk (HM) components may influence infant growth and development. This study aimed to investigate relationships between infant body composition (BC) and HM lactose, insulin, and glucose (concentrations and calculated daily intakes (CDI)) as well as 24-h milk intake and maternal BC at 3 months postpartum. HM samples were collected at 2 months postpartum. Infant and maternal BC was assessed with bioimpedance spectroscopy. Statistical analysis used linear regression accounting for infant birth weight. 24-h milk intake and CDI of lactose were positively associated with infant anthropometry, lean body mass and adiposity. Higher maternal BC measures were associated with lower infant anthropometry, z-scores, lean body mass, and adiposity. Maternal characteristics including BC and age were associated with concentrations and CDI of HM components, and 24-h milk intake. In conclusion, 24-h intake of HM and lactose as well as maternal adiposity are related to development of infant BC.

## 1. Introduction

Human milk (HM) is typically the sole food source for the infants in the first 6 months of life, through which they acquire all elements necessary for their growth and development [1,2]. HM provides a constant supply of micro- and macronutrients, bioactive molecules including carbohydrates (lactose and glucose) and hormones (insulin) to the infant during early life, ensuring appropriate nutrition, maturation, and protection against infection and growth [3,4,5]. These components may also affect infant gut microbiota, appetite regulation and metabolism, as well as infant body composition (BC). Development of infant BC in the early months postpartum is known to play a significant role in the programming of obesity later in life [6,7], but little is known as to how the predominant HM carbohydrate lactose, its precursor glucose, and glucose uptake regulator insulin influence the development of BC during infancy. 

Lactose is the least variable macronutrient in HM across the first 12 months of lactation [8,9], with estimated levels of approximately 67–78 g/L [5]. Lactose has been shown to upregulate gastrointestinal expression of the antimicrobial peptide cathelicidin in epithelial and monocytic cells, which may provide protection against pathogens and influence infant gut microbiota [10]. In addition, lactose can act as an energy source for Bifidobacterium spp. and Lactobacillus spp. [11], which are the dominant bacterial species found in the gut of the breastfed infant [12], indicating a potential effect on infant growth. There are limited studies investigating the effect of HM lactose on infant BC, with the conflicting results. Two previous studies have shown that the concentration of HM lactose (4–8 weeks lactation) was positively related to infant weight, BMI and adiposity gains between 3 and 12 months [13] and positively associated with an increasing weight-for-length z-score (WLZ) at 6-months among infants breastfed by mothers with overweight status and obesity [14]. However, another study reported that HM lactose concentration was not related to infant BC or anthropometrics during the first 12 months of lactation, yet higher calculated daily intakes (CDI) of lactose were associated with greater infant adiposity and decreased lean mass, suggesting that lactose could contribute to the development of BC in breastfed infants [6]. 

Insulin is produced by beta cells in the pancreas that facilitate glucose uptake and regulate carbohydrate, lipid and protein metabolism [15,16]. HM insulin concentrations change over the course of lactation, with lowest concentration reported in colostrum, followed by observed increases in mature milk [17]. However, its function with respect to infant growth and development of BC has not been firmly established. Existing studies describing the potential impact of HM insulin on infant health present conflicting results. One study reported no association between 1-month HM insulin concentration and infant body length, total fat mass (FM), %FM, and trunk FM at 6 months [18]. A second study showed HM insulin concentration was negatively associated with infant weight, WLZ, BMI-for-age z-score (BMIAZ), and lean mass in infants from both mothers with normal weight and mothers with obesity at 1 month of age [19]. Yet a third study reported a U-shaped association between HM insulin concentration and infant z-scores [20]. The mixed evidence of HM insulin association with infant BC provides impetus for further investigations.

Glucose is a less abundant carbohydrate found in HM with an average concentration of 255.2 ± 75.3 μg/mL over the first 6 months of lactation [21]. Glucose is a precursor for lactose [22] and its concentration in HM is proportional to the rate of milk secretion [23] and indicative of milk supply [24]. However, HM glucose concentration is also shown to be higher in mothers with obesity and overweight status compared to women of normal weight [25], and may therefore influence infant growth and health outcomes. A recent study showed that HM glucose concentration was positively associated with infant WLZ and BMIAZ, but not FM or lean mass of breastfed infants [19]; in contrast, two other studies found no association between concentration of HM glucose and infant BC [18,21]. Evidence regarding the influence of HM glucose on infant growth is scarce and requires further elucidation. 

HM lactose, insulin and glucose may contribute to infant growth; however, the literature on the influence of HM components concentrations, and particularly intakes, on infant BC development is sparse. The aim of this cross-sectional study was to assess relationships of these HM components with measures of infant growth and BC in healthy term 3-month-old exclusively breastfed infants. In addition, relationships with maternal BC and 24-h milk intake were explored.

## 2. Materials and Methods

### 2.1. Study Design

Pregnant women were recruited from the community and existing online networks during the third trimester of pregnancy (>30 weeks gestation) to participate in a birth cohort study. The BLOSOM cohort (Breastfeeding Longitudinal Observational Study of Mothers and kids) is a single-centre prospective cohort being conducted in Perth (Western Australia) designed to elucidate the mechanisms by which HM impacts infant growth, body composition and health. For the present study, the analysis was limited to dyads that provided milk samples and/or measured 24-h milk intakes and BC and anthropometrics measurements were available at 3 months postpartum (n = 67). Inclusion criteria were women self-reported as healthy with no major pregnancy complications, intention to exclusively breastfeed up to at least five months postpartum, and to breastfeed until 12 months postpartum. Exclusion criteria included infant factors that could potentially influence growth and development of BC, maternal smoking, pregnancy complications such as preterm labor and gestational diabetes mellitus (GDM). All mothers provided informed written consent to participate in the study, which was approved by the Human Research Ethics Committee at The University of Western Australia (RA/4/20/4023).

### 2.2. Sample and Health Data Collection

HM samples were collected by mothers at 2 months postpartum. Mothers elected one breast from which to donate the HM sample. Mothers were asked not to breastfeed or express milk from the elected breast for at least two hours prior to sample collection. Mothers washed their hands thoroughly with soap and water and wore disposable nitrile powder-free gloves (Complete Office Supplies, NSW, Australia) during sample collection. The nipple and areola of the expressing breast were cleaned with alcohol and chlorhexidine prep pads (70% isopropyl alcohol and 2% chlorhexidine digluconate, Reynard Health Supplies, NSW, Australia), followed by rinsing with sterile saline solution (Livingstone, NSW, Australia) and drying with sterile gauze swabs (Livingstone, NSW, Australia). Up to 20 mL (otherwise as much as possible) of HM was hand-expressed directly into sterile tubes (Greiner Bio-One, Kremsmünster, Austria). HM samples were stored at 4 °C in the fridge at the home of the participant before being collected within 6–24 h and transported to the laboratory on ice, where they were immediately aliquoted into sterile tubes (Sarstedt, Numbrecht, Germany) and stored at −80 °C until further analysis.

Mothers answered a background questionnaire at the time of recruitment, and an infant and maternal questionnaire on sample collection day.

### 2.3. Biochemical Analysis

HM aliquots were thawed for 1 h at room temperature. Whole milk samples were used for measuring insulin and skim milk samples were used for measuring glucose and lactose concentrations. HM components were measured in duplicate. To acquire skim HM, samples were defatted by centrifugation in glass capillary tubes (Kimble Chase, Rockwood, TN, USA) at room temperature at 12,000× g for 10 min [26]. The fat layer was removed by cutting the tubes below the bottom of the lipid layer and discarded. 

Insulin concentrations in whole HM samples were analyzed using the Human Insulin ELISA kit (BioVendor, Brno, Czech Republic) according to the instructions of the manufacturer. A six-point standard curve ranging from 0–250 µIU/mL was constructed. The insulin detection limit was 0.26 µIU/mL. The results obtained for controls were within the range specified by the manufacturer.

Glucose concentration in skim HM samples (diluted in 0.1 M K-Phosphate buffer, 1:2) was analyzed by enzymatic assay. Briefly, skimmed HM samples, standards and controls were pipetted (5 µL) into wells on a 96-well microtiter plate and 200 µL of the reagent cocktail (9.6 U/mL glucose oxidase, 2.5 U/mL peroxidase, reduced ABTS (2,2-azino-di-(3-ethyl-benzthiazolinsulfonate)-6-sulfonate) and 0.1 M-phosphate buffer, pH 7.2) was added to each well. The plates were incubated for 5 min at room temperature in the dark. Absorbance was measured at 405 nm on a plate spectrophotometer (Enspire Multimode Plate Reader, Waltham, MA, USA). A seven-point standard curve ranging from 0–2 mmol/L was constructed. The glucose detection limit was 0.07 mmol/L, and the inter-assay CV was 11.85%. Recovery of a known amount of glucose added to the milk samples was 98% ± 1.4% (n = 9). The results were converted into g/L using the molecular weight of glucose (180.16 g/mol). 

Lactose concentration in skim HM samples (diluted in distilled de-ionized water, 1:150) was analyzed with the lactose colorimetric enzymatic assay according to the protocol as previously described by Mitoulas et al. [9]. A seven-point standard curve ranging from 0–300 mmol/L was constructed. The detection limit was 0.51 mmol/L, and the inter-assay CV was 4.23%. Recovery of a known amount of lactose added to the milk samples was 101% ± 3.14% (n = 8). The results were converted into g/L using the molecular weight of lactose (360.3 g/mol).

### 2.4. Twenty-Four Hour Milk Intake

Infant 24-h milk intake was measured at 3 months postpartum (3.3 ± 0.4 months; range: 2.2–4.2) by mothers in their homes using the 24-h milk profile protocol as described previously [27]. Briefly, this involved mothers weighing their infant before and after each breastfeed on the electronic Baby Weigh Scales (Medela Inc., McHenry, IL, USA, resolution 2 g, accuracy ±0.034%). HM intake (g) was calculated by subtracting the weight of the infant before the feed from the weight after the feed. 3-month 24-h milk intakes were considered representative of intakes during the exclusive breastfeeding period as there is no significant variation in HM intake from 1 to 6 months within infants [28]. 

### 2.5. Calculated Daily Intakes of HM Components

Calculated daily intakes (CDI) of HM components (g) were determined as the concentration of the component (g/L) multiplied by 24-h intake that were converted from g to mL using the density of HM of 1.03 g/mL [29]. 

### 2.6. Anthropometry and Body Composition Measurements

Anthropometric and BC measurements were performed for both mothers and infants at 3 months postpartum (3.1 ± 0.1 months; range: 2.9–3.5). Maternal weight was measured using Seca electronic scales (±0.1 kg; Seca, Chino, CA, USA). Height was self-reported by mothers or measured against a marked wall. Infant clothes were removed before measuring weight using electronic scales (Medela Inc.). Infant crown to heel length was measured in supine position with an infantometer (Seca, Chino, CA, USA). Infant head circumference was measured with non-stretch tape to the nearest 0.1 cm. Infant z-scores (WLZ), weight-for-age z-scores (WAZ), length-for-age z-scores (LAZ), BMIAZ and head circumference-for-age z-scores (HCAZ) were measured using the World Health Organization (WHO) Anthro software v3.2.2 [30]. 

Maternal and infant BC was measured with bioelectrical impedance spectroscopy using a battery-operated bioelectrical impedance analyser Impedimed SFB7 (ImpediMed, Brisbane, QLD, Australia) according to the protocols described previously by Gridneva et al. [31]. In addition to standard BC measurements (fat-free mass (FFM), FM, and %FM), the height-normalized BC indices of mothers and infants (FFMI, FMI) as well as FM to FFM ratio (FM/FFM) were calculated as described previously [6]. 

### 2.7. Statistical Analysis

Eleven groups of analyses were performed on these cross-sectional data using linear regression, all with a cofounder of infant birth weight. 

Groups one to four had infant anthropometric and BC measures as response variables taken at 3 months postpartum (weight, length, BMI, head circumference, FFM, FFMI, FM, FMI, %FM, FM/FFM and z-scores). Group one had explanatory variables of infant sex, gestational age, birth weight and 24-h milk intake; group two had explanatory variables based on milk concentrations of lactose, insulin and glucose; group three had explanatory variables based on CDI of lactose, insulin and glucose; group four were maternal factors (birth mode and BC measures; weight, BMI, FFM, FFMI, FM, %FM, FMI, FM/FFM). 

Groups five to seven had infant 24-h milk intake as a response. Group five had explanatory variables of infant sex, gestational age and birth weight; group six had explanatory variables based on CDI of lactose, insulin and glucose; group seven had maternal explanatory variables. 

Groups eight to ten had CDI of lactose, insulin and glucose as response variables. Group eight had a single explanatory variable of infant 24-h milk intake; group nine was based on HM concentration of lactose, insulin and glucose; group ten were maternal factors. 

Group eleven response variables were based on concentrations of lactose, insulin and glucose; explanatory variables are maternal factors.

Descriptive statistics were reported as mean ± standard deviation (SD) and range/percentage; and modeling results as parameters estimates ± standard error (SE). Missing data were dealt with using available case analysis. Statistical analyses were performed in R 4.0.2. The significance level for this investigative study was set at the 5% level. Due to the investigative nature of the study, p-values were not adjusted for multiple comparisons. The results should be interpreted in terms of the occurring patterns instead of focusing on the single significant test results.

## 3. Results

### 3.1. Participants Characteristics

Sixty-seven mother-infant pairs were recruited; ten were excluded due to formula use (n = 2), absence of BC and 24-h milk intake measurements (n = 1), low birth weight (n = 1), 24-h milk intake measured outside of the time point of study (n = 2), or low 24-h milk intake (<500 mL/day) (n = 4). Missing data included maternal weight, BMI and BC measurements (n = 5), infant weight and length measurements, WLZ, WAZ, LAZ, BMI and BMIAZ (n = 5), infant BC measurements (n = 7), infant head circumference and HCAZ (n = 10), and 24-h milk intake and CDI of lactose, insulin, and glucose (n = 12). The characteristics of the participants (n = 57) are shown in Table 1. 

### 3.2. Human Milk Components 

HM components concentrations and CDI are presented in Table 2. 

### 3.3. Maternal and Infant Body Composition

Maternal and infant BC measurements and infant anthropometric z-scores at 3 months postpartum are presented in Table A1.

Mothers (*n* = 52) were classified as being either underweight (BMI < 18.5, 3.9%, *n* = 2; %FM < 21, 0%, *n* = 0), with normal weight (BMI 18.5–24.9, 40.4%, *n* = 21; %FM 21–32.9, 32.7%, *n* = 17), with overweight status (BMI 25–29.9, 26.9%, *n* = 14; %FM 33–38.9, 34.6%, *n* = 18) or with obesity (BMI > 30, 28.9%, *n* = 15; %FM > 39, 32.7%, *n* = 17) [32]. 

Infant birth weight was positively associated with 3-month anthropometry, lean mass, adiposity, and z-score measurements. Additionally, infant gestational age was positively associated with LAZ (Table A2). 

After accounting for infant birth weight, males were heavier, longer, with higher BMI and larger head circumference than females. Additionally, males had higher FFM, FFMI, and lower %FM and FM/FFM ratio compared to female infants (Table A2).

Associations between maternal BC and infant anthropometrics, BC measures and *z*-scores are detailed in Table A3. 

Higher maternal weight, BMI, FM, %FM, FMI, FFM and FFMI, were associated with lower infant anthropometric measures (weight, BMI, and head circumference). Similarly, higher maternal age and BC measures were associated with lower infant lean body mass (FFM and FFMI), adiposity (FM, %FM, FMI and FM/FFM) and *z*-scores (HCAZ, LAZ, WAZ, WLZ and BMIAZ) (Table A3). 

### 3.4. Twenty-Four Hour Milk Intake and HM Components

Higher concentrations of all three HM components resulted in higher CDI (lactose: 0.817 ± 0.284 g/day, *p* = 0.006; insulin: 0.692 ± 0.034 ng/day, *p* < 0.001; glucose: 0.880 ± 0.080 g/day, *p* < 0.001); whilst higher 24-h milk intakes resulted in higher CDI of lactose (0.085 ± 0.006 g/day, *p* < 0.001) and glucose (0.0003 ± 0.0001 g/day, *p* < 0.001) but not insulin (0.0004 ± 0.0007 ng/day, *p* = 0.53).

### 3.5. Twenty-Four Hour Milk Intake, HM Components, and Infant Body Composition

Significant associations between 24-h milk intake, CDI of HM components and infant BC are detailed in Table 3. 

Higher CDI of lactose was associated with higher infant anthropometry (weight and length), adiposity (FM and FMI), lean body mass (FFM and FFMI) and WAZ (Table 3). 

Concentrations and CDI of insulin and glucose were not associated with infant anthropometry and BC measurements (data not shown), except for CDI of glucose which was positively associated with infant head circumference.

Higher 24-h milk intake was associated with higher infant anthropometry (weight and length), lean body mass (FFM and FFMI), adiposity (FM and FMI) and WAZ (Table 3).

### 3.6. Maternal Characteristics and Human Milk Components

Associations between maternal characteristics and HM components and 24-h milk intake are detailed in Table 4.

CDI of lactose and 24-h milk intake were negatively associated with maternal weight, BMI, lean body mass (FFM, and FFMI) and adiposity (FM and FMI). No significant association with maternal adiposity was seen for either CDI of insulin or glucose.

HM insulin concentration was positively associated with maternal BMI and adiposity (FMI) and negatively with age (Table 4). 

## 4. Discussion

**Figure 1:**
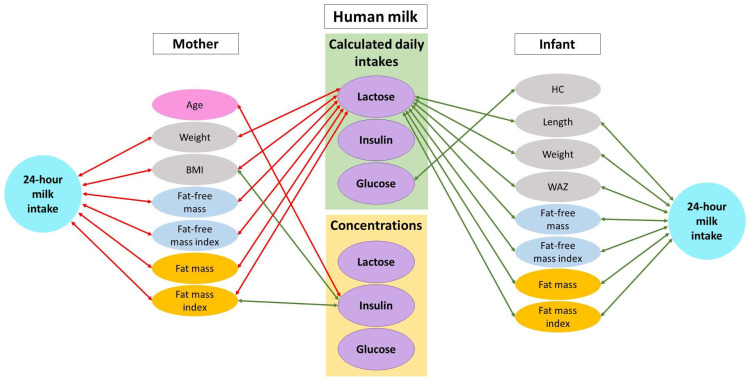
Possible lactocrine programming of the infant body composition as researched. Green arrows indicate positive associations and red arrows—negative associations. BMI—body mass index; HC—head circumference; WAZ—weight-for-age z-score.

HM is a highly complex, biological fluid that has long-term beneficial health effects for infants [3,4,33]. This study sheds new light on the complex mechanisms by which HM components may influence infant BC. In this small investigative study, we found that at 3 months postpartum HM insulin and glucose concentrations and CDI were not associated with infant BC, whereas 24-h milk intake and CDI of HM lactose were positively associated with infant lean and fat mass as well as anthropometry. Furthermore, negative associations between maternal BC and infant 24-h milk intake and BC measurements were observed. Together these results emphasize the critical role HM components may play in programming infant growth and development in the early life (Figure 1).

In this cohort of healthy mothers and exclusively breastfed infants, we observed positive associations between CDI of lactose and infant anthropometry, lean body mass, adiposity and WAZ (Table 3; Figure 1). This emphasises the importance of measuring daily intakes, in addition to concentrations, of HM components. These results are similar to two previous studies that also analyzed associations between lactose intake and infant BC measures. In a smaller cohort Gridneva et al. reported higher CDI of lactose was associated with infant whole body adiposity (increased FM, FMI, %FM, FM/FFM ratio, BMI and decreased 12-month FFMI) [6] as well as higher subcutaneous-abdominal fat areas [34], and Butte et al. reported that daily intake of lactose was positively correlated with infant weight and FFM gain but not with FM or % FM, at 3 and 6 months [35]. Lactose is the major HM carbohydrate [36], providing a substantial proportion of energy to the infant, thus it is biologically plausible that the current and previous studies show the potential of HM lactose to promote weight gain and healthy fat deposition in the infant and thereby influence long-term health. 

Contrary to intake, we observed no relationships between HM lactose concentration and infant anthropometry and BC measures. Our results are supported by four previous studies that reported no association between lactose concentration and infant anthropometry and BC measured during the first 12 months of lactation [6,34], and in the first 6 months in exclusively breastfed infants [9,21]. However, two other studies have reported significant results. One study showed that lactose concentration at 2–4 weeks lactation was positively associated with increased WLZ at 6 months, but only among infants of women with overweight status and obesity [14]. Another reported that HM % lactose was positively related to infant weight, BMI and adiposity gains between three and 12 months [13]. Lactose concentration remains stable between 1 and 6 months of lactation [37], therefore, the lack of association between lactose concentration and infant adiposity may be explained by its role in maintaining osmotic pressure in HM [38]. Further work is required to better understand the roles of HM lactose in the breastfed infant. 

We observed no relationships between HM insulin concentration and CDI and 3-month infant anthropometry and BC measures, despite the presence of mothers with obesity (*n* = 15) and overweight status (*n* = 14). This is similar to two previous studies that showed no association between HM insulin 1- and 6-month concentrations and infant BC measures [18,21]. However, a third study reported negative associations with infant weight, WLZ, BMIAZ, and lean mass [19], and a fourth study reported a U-shaped association between HM insulin concentration and infant BC, with intermediate concentrations predicting lower WLZ and BMI for age z-scores (BMIAZ) at 4 and 12 months [20]. The contrasting results are likely due to different study designs [20], and different methods used for assessing infant BC [19]. Alternatively, the lack of associations of HM insulin concentration and CDI with infant BC could be due to the central role of insulin in glucose metabolism [39] and as regulator of food intake and energy balance [40], or interactions with other hormones, such as leptin [41].

In our study HM glucose concentration and CDI were not related to infant lean body mass or adiposity. This is in contrast to a cross-sectional study [19], that reported that HM glucose concentrations were positively associated with 1-month infant WLZ and BMIAZ. Whereas a longitudinal study, ref. [21] reported no significant associations with infant *z*-scores. The differences between the studies could be due to differences in design and statistical methods. As glucose is a relatively small contributor to HM carbohydrate content [42] and acts as a universal and essential fuel in energy metabolism [22], it is possible that HM glucose may not directly modulate infant lean body mass and adiposity, but instead act as the principal stimulus for insulin secretion which in turn facilitates diffusion of glucose into fat and muscle cells [15], affecting infant growth. Interestingly, glucose CDI was positively associated with infant head circumference. Head circumference is regarded as a proxy measurement for infant brain size [43] and correlates with cognitive function and intracranial volume [44,45]. In 2- and 5-month old infants an increase in glucose metabolic activity in several brain regions was related to their functional maturation [46], which may also reflect on the head circumference. However, further work is required to confirm this finding of the present study.

HM intake and duration of exclusive breastfeeding are known to relate to infant growth rate [9,47,48,49,50]. We found positive associations between 24-h milk intake and infant anthropometry (weight and length), lean body mass (FFM and FFMI), adiposity (FM and FMI) and *z*-scores (WAZ) (Table 3; Figure 1). Similarly, two previous studies have reported positive association between HM intake and infant growth during the first year of life. In the DARLING study, 3-month HM intake was positively associated with weight at 3 months [47]. However, the relationships between 24-h milk intake and infant BC were reported to be differential in the second study [31]; a positive association was found with adiposity and a negative association with lean mass. These data suggest that 24-h milk intake influences BC development in addition to supporting the nutritional needs of the rapidly growing infant. 

Our study also found strong positive associations between infant birth weight and anthropometry, lean body mass, adiposity, and z-score measurements, similar to previous studies that showed positive associations with FM and FMI [31,51]. Given that increased birth weight may predispose infants to obesity and type 2 diabetes mellitus later in life [52,53], it is important to understand the relationship between infant birth weight and BC. 

Our results showed that male infants, as expected, displayed higher anthropometry (weight, length, BMI and head circumference) and lean mass (FFM and FFMI) as well as lower adiposity (%FM and FM/FFM; Table A2). This is in line with previous studies which showed greater FFM [31,35,54,55] and lower %FM [56] in male than in female infants. However, some studies have presented contrary results, suggesting no differences in %FM between sexes [31,57]. The differences between the studies could be due to the use of different methods to assess BC such as air-displacement plethysmography [57], bioelectrical impedance spectroscopy [31], or small to modest sample size. The effect of infant sex on adiposity and HM composition during early life is still debated, BC developmental differences between infant sexes could be partially explained by sex-specific needs for early nutrition and differences in infants’ hormones. However, limited human studies show contradictory results regarding differences in HM composition for infant sex [58,59,60,61], requiring further validation and clarification of sex-specific differences in HM composition and their impact on infant growth trajectories accounting for milk intake. 

In this study, we also investigated associations between maternal characteristics/adiposity and HM components. Higher maternal BMI and FMI were associated with higher HM insulin concentration (Table 4) and this is consistent with findings from previous studies [17,20,25,62], with the exception of one [63]. It has been shown that mothers with overweight status and obesity have a higher concentration of HM insulin compared to mothers of normal weight [25,64], suggesting maternal adiposity may play a role in regulation of HM insulin concentration. Whilst the underlying mechanisms involved are not yet fully elucidated, obesity is associated with increased insulin secretion independently of insulin resistance [65] and insulin is transported into HM at concentrations comparable to serum [66], suggesting either an active transport mechanism or a passive diffusion from serum. Furthermore, maternal age in this cohort was negatively associated with HM insulin (Table 4) which is in line with decreased insulin secretion observed with age, even after adjustments for adiposity and physical activity [67], again supporting active/passive transport.

A novel finding of this study is that maternal BC was not associated with either glucose concentration, or CDI (data not shown). In contrast, one previous study showed that mothers with overweight and obesity status had higher HM glucose concentrations compared to mothers of normal weight, and that higher concentrations were inter-correlated with pre-pregnancy BMI [25]. Research in this area is, however, extremely limited and further work is needed to validate the present findings and to discern the mechanisms by which maternal BC may affect HM glucose.

We found no significant association between maternal adiposity and HM lactose concentration, similar to previous studies from Western populations [6,68,69,70]. However, two studies in Asian populations reported a negative association between HM lactose concentration and maternal BMI [71,72]. The differences between studies may be due to differences in maternal BMI as participants in Asian studies had a lower BMI range compared to those in the Western studies, the use of different statistical methods, or due to genetic/dietary differences between populations. Contrary to lactose concentration, CDI of lactose, as well as 24-h milk intake, were negatively related to maternal weight, BMI, and BC measures suggesting the intakes are driven either via the maternal HM production and/or infant demand. Similarly, a recent study showed a negative relationship between maternal FM and HM intake [73], supporting the notion that maternal characteristics may influence the volume of HM consumed by the infant. To date, only one study has analyzed the relationship between maternal BC and CDI of lactose [6], reporting no associations, however, this study was based on a small sample size (*n* = 20).

The current study is one of few to investigate relationships between maternal BC and infant BC measurements during exclusive breastfeeding. Previous studies, based on maternal BMI [74,75,76,77], anthropometry [78] and BC measured with bioelectrical impedance analysis during pregnancy [79], reported positive associations with infant birth weight, FM, and %FM. Our study showed no associations between maternal BC measures and infant %FM, however maternal BMI and BC measures were negatively associated with infant anthropometry (weight, head circumference and BMI), adiposity (FM and FMI), lean mass (FFM, FFMI), and *z*-scores (WAZ, WLZ, BMIAZ and HCAZ) (Table A3). In line with our results, a small longitudinal study reported that increased maternal adiposity was related to lower infant FFM over the course of the first year of lactation [31], and a cross-sectional study showed that increased maternal BMI was associated with lower infant FFM in infants under one month of age [76]. This suggests that early life BC changes may be influenced by maternal BC status. Therefore, maintaining healthy maternal adiposity pre-pregnancy and during lactation may be beneficial for the development of infant lean and FM [31]. 

This small investigative study has focused on exclusively breastfed infants; thus, it is reflective of the influence of HM components on the development of infant BC. The strengths of this study include assessment of maternal and infant BC as well as 24-h milk intake and CDI of HM components. However, it should be noted that the study includes some limitations, such as the cross-sectional nature of the study, the moderate sample size and no access to the gold standard BC method or ability to account for maternal dietary intake. Our population consisted of term, healthy, exclusively breastfed infants from predominantly Caucasian mothers of higher social-economic status; therefore, the results may not be transferable to other populations.

## 5. Conclusions

The findings from this investigative study demonstrate that increased CDI of lactose and 24-h milk intake relate to increased infant growth and BC measures during the first 3 months of life, whilst concentrations and CDI of HM insulin and glucose do not relate to infant BC development. Additionally, maternal lean mass and adiposity influence infant 24-h milk intake as well as infant BC development. These findings highlight the importance of daily intakes, not just concentrations, of HM components and have implications for infant BC development and potentially health later in life.

## Figures and Tables

**Table 1:** Maternal and infant characteristics.

Characteristics	Mean ± SD (Min–Max) or n (%)
Maternal age at infant birth (years)	32.7 ± 4.6 (25.1–46.4)
Maternal ethnicity	
Caucasian	48 (84.2)
Asian	5 (8.8)
Other	4 (70)
Parity	2.1 ± 0.8 (1–4)
Mode of delivery	
Vaginal	36 (63.2)
Elective caesarean section	14 (24.6)
Emergency caesarean section	7 (12.3)
Maternal height (cm)	164.7 ± 6.9 (148.0–178.0)
Maternal weight (kg)	72.8 ± 15.3 (47.5–119.8) ^a^
Infant gestational age (weeks)	39.3 ± 1.1 (37.0–41.2)
Infant sex	
Male	27 (47.4)
Female	30 (52.6)
Infant birth weight (kg)	3.505 ± 0.413 (2.610–4.705)
Infant birth length (cm)	50.8 ± 2.1 (46.0–55.0)
Infant weight (kg)	6.150 ± 0.756 (4.638–8.410) ^a^
Infant length (cm)	61.1 ± 2.6 (55.5–67.0) ^a^
Infant head circumference (cm)	40.8 ± 1.2 (38.0–43.0) ^b^
24-h milk intake (mL)	792.8 ± 176.1 (511.7–1304.9) ^c^

Data are mean ± SD and ranges or percentage. All anthropometrics were measured at 3 months postpartum unless otherwise stated. ^a^ *n* = 52, ^b^  *n* = 47, ^c^  *n* = 45.

**Table 2:** Human milk components presented as concentrations and 24-h intakes.

Components	Concentration ^a^ Mean ± SD (Min–Max)	CDI ^b^ Mean ± SD (Min–Max)
Lactose	86.56 ± 7.91 (g/L) (69.91–106.09)	68.35 ± 16.63 (g/day) (39.91–129.27)
Insulin	1.48 ± 1.14 (ng/mL) (0.46–5.90)	1.16 ± 0.78 (ng/day) (0.30–4.17)
Glucose	0.26 ± 0.09 (g/L) (0.09–0.47)	0.20 ± 0.09 (g/day) (0.05–0.49)

Data are mean ± SD and ranges. ^a^  *n* = 57, ^b^  *n* = 45, CDI—calculated daily intakes based on 24-h milk intake.

**Table 3:** Associations between infant body composition and 24-h milk and lactose intakes at 3 months postpartum.

Predictor	Intercept	SE	Slope	SE	Predictor	Infant Birth Weight
					*p*-Value	*p*-Value
Infant weight (kg)						
Lactose intake (g/day)	1.779	0.643	0.017	0.005	0.001	<0.001
24-h milk intake (mL)	1.672	0.638	0.002	0.0004	<0.001	<0.001
Infant length (cm)						
Lactose intake (g/day)	46.010	2.345	0.047	0.017	0.010	<0.001
24-h milk intake (mL)	45.905	2.388	0.004	0.002	0.016	<0.001
Head circumference (cm)						
Glucose intake (g/day)	35.338	1.495	4.304	1.885	0.028	0.004
Infant fat-free mass (kg)						
Lactose intake (g/day)	1.841	0.440	0.011	0.003	0.001	<0.001
24-h milk intake (mL)	1.776	0.438	0.001	0.0003	<0.001	<0.001
Infant fat-free mass index (kg/m^2^)						
Lactose intake (g/day)	9.757	1.257	0.026	0.009	0.009	0.011
24-h milk intake (mL)	9.591	1.252	0.003	0.001	0.005	0.013
Infant fat mass (kg)						
Lactose intake (g/day)	−0.081	0.267	0.005	0.002	0.011	<0.001
24-h milk intake (mL)	−0.118	0.266	0.001	0.0002	0.006	<0.001
Infant fat mass index (kg/m^2^)						
Lactose intake (g/day)	0.956	0.815	0.014	0.006	0.028	<0.001
24-h milk intake (mL)	0.855	0.813	0.001	0.001	0.017	<0.001
Weight-for-age z-score						
Lactose intake (g/day)	−5.200	0.792	0.017	0.006	0.007	<0.001
24-h milk intake (mL)	−5.330	0.785	0.002	0.001	0.003	<0.001

Data are parameter estimate ± standard error of measurement (SE) after accounting for infant birth weight.

**Table 4:** Associations between human milk components, 24-h milk intakes and maternal characteristics at 3 months postpartum.

Maternal Predictor	Intercept	SE	Slope	SE	Predictor	Infant Birth Weight
					*p*-Value	*p*-Value
Calculated daily intakes of HM components						
Lactose (g/day)						
Weight (kg)	47.115	19.137	−0.462	0.163	0.007	0.009
BMI (kg/m^2^)	55.258	19.195	−1.501	0.459	0.002	0.007
FFM (kg)	50.552	20.228	−0.775	0.327	0.023	0.014
FM (kg)	39.098	19.043	−0.677	0.268	0.016	0.020
FFMI (kg/m^2^)	63.565	20.691	−3.000	0.987	0.004	0.007
FMI (kg/m^2^)	42.531	19.061	−1.909	0.721	0.011	0.024
Concentrations of HM components						
Insulin (ng/mL)						
Age (years)	2.392	1.666	−0.073	0.032	0.027	0.24
BMI (kg/m^2^)	−1.090	1.389	0.067	0.032	0.044	0.55
FMI (kg/m^2^)	−0.543	1.325	0.100	0.048	0.043	0.42
24-h milk intake (mL)						
Weight (kg)	552.379	197.950	−5.256	1.688	0.003	0.004
BMI (kg/m^2^)	630.592	201.169	−16.005	4.811	0.002	0.005
FFM (kg)	590.075	210.553	−8.743	3.403	0.014	0.008
FM (kg)	461.557	197.438	−7.740	2.781	0.008	0.011
FFMI (kg/m^2^)	714.516	217.715	−31.497	10.383	0.004	0.005
FMI (kg/m^2^)	495.925	199.567	−20.608	7.548	0.009	0.016

Data are parameter estimate ± standard error of measurement (SE) after accounting for infant birth weight. BMI—body mass index; FFM—fat-free mass; FFMI—fat-free mass index; FM—fat mass; FMI—fat mass index; HM—human milk.

## Data Availability

The data presented in this study are available from the corresponding author upon reasonable request.

## Appendix A

**Table A1:** Maternal and infant body composition at 3 months postpartum.

Body Composition	Infant (Male) Mean ± SD (Min–Max)	Infant (Female) ^d^ Mean ± SD (Min–Max)	Infant (Pooled) Mean ± SD (Min–Max)	Mother ^f^ Mean ± SD (Min–Max)
Fat-free mass (kg)	4.92 ± 0.46 (3.80–6.01) a	4.27 ± 0.27 (3.83–4.76)	4.57 ± 0.49 (3.80–6.01) e	45.68 ± 8.46 (18.00–70.89)
Fat-free mass index (kg/m^2^)	17.78 ± 1.03 (13.70–17.93) a	14.19 ± 0.67 (12.93–15.41)	14.92 ± 1.17 (12.93–17.93) e	16.85 ± 2.79 (6.30–23.96)
Fat mass (kg)	1.65 ± 0.35 (0.84–2.50) a	1.51 ± 0.24 (1.06–1.94)	1.58 ± 0.30 (0.84–2.50) e	26.75 ± 9.42 (11.25–53.93)
Fat mass index (kg/m^2^)	5.28 ± 0.98 (3.02–7.47) a	5.01 ± 0.70 (3.80–6.22)	5.14 ± 0.84 (3.02–7.47) e	9.88 ± 3.41 (3.94–18.99)
Fat mass (%)	24.88 ± 2.49 (18.05–29.76) a	25.99 ± 1.96 (21.52–29.04)	25.48 ± 2.27 (18.05–29.76) e	35.91 ± 6.16 (21.88–53.35)
Fat mass to fat-free mass ratio	0.33 ± 0.04 (0.22–0.42) a	0.35 ± 0.04 (0.27–0.41)	0.34 ± 0.04 (0.22–0.42) e	0.58 ± 0.16 (0.37–1.14)
Body mass index (kg/m^2^)	16.97 ± 1.32 (15.06–19.92) b	15.98 ± 1.08 (14.05–17.99)	16.46 ± 1.29 (14.05–19.92) f	26.88 ± 5.21 (18.00–40.95)
Infant z-scores				
Weight-for-length z-score	0.04 ± 0.92 (−1.23–2.54) b	−0.22 ± 0.83 (−1.82–1.11)	−0.09 ± 0.89 (−1.82–2.54) f	
Weight-for-age z-score	0.19 ± 1.03 (−2.79–2.49) b	−0.15 ± 0.71 (−1.64.−1.15)	0.01 ± 0.89 (−2.79–2.49) f	
Length-for-age z-score	0.32 ± 1.31 (−3.09–2.83) b	0.11 ± 1.11 (−2.25–2.18)	0.21 ± 1.20 (−3.09–2.83) f	
BMI-for-age z-score	0.10 ± 0.90 (−1.39–1.95) b	−0.28 ± 0.72 (−1.63–1.05)	−0.10 ± 0.82 (−1.63–1.95) f	
Head circumference-for-age z-score	0.55 ± 0.93 (−1.34–2.08) c	0.67 ± 0.90 (−1.07–2.56) b	0.62 ± 0.91 (−1.34–2.56) g	

Data are mean ± SD and ranges. ^a^  *n* = 23, ^b^  *n* = 25, ^c^  *n* = 22, ^d^  *n* = 27, ^e^  *n* = 50, ^f^  *n* = 52, ^g^  *n* = 47. BMI—body mass index.

**Table A2:** Associations between infant characteristics and body composition at 3 months postpartum.

Infant Predictor	Intercept	SE	Slope	SE	Predictor	Infant Birth Weight
					*p*-Value	*p*-Value
Infant weight (kg)						
Sex (male)	2.259	0.561	0.638	0.137	<0.001	<0.001
Birth weight (kg)	2.143	0.666	0.001	0.0002	<0.001	NA
Infant length (cm)						
Sex (male)	47.669	2.208	1.459	0.540	0.010	<0.001
Birth weight (kg)	47.405	2.340	0.004	0.001	<0.001	NA
Infant body mass index (kg/m^2^)						
Sex (male)	13.434	1.343	0.892	0.329	0.009	0.060
Birth weight (kg)	13.272	1.425	0.001	0.0004	0.029	NA
Infant head circumference (cm)						
Sex (male)	36.160	1.350	0.684	0.298	0.027	0.003
Birth weight (kg)	36.176	1.413	0.001	0.0004	0.002	NA
Infant fat-free mass (kg)						
Sex (male)	2.271	0.323	0.559	0.080	<0.001	<0.001
Birth weight (kg)	2.104	0.454	0.001	0.0001	<0.001	NA
Infant fat-free mass index (kg/m^2^)						
Sex (male)	10.763	0.863	1.433	0.215	<0.001	<0.001
Birth weight (kg)	10.332	1.188	0.001	0.0003	<0.001	NA
Infant fat mass (kg)						
Birth weight (kg)	−0.062	0.264	0.001	0.0001	<0.001	NA
Infant fat mass index (kg/m^2^)						
Birth weight (kg)	0.967	0.779	0.001	0.0002	<0.001	NA
Infant fat mass (%)						
Sex (male)	15.374	2.073	−1.616	0.516	0.003	<0.001
Birth weight (kg)	15.860	2.249	0.003	0.001	<0.001	NA
Infant fat mass to fat-free mass ratio						
Sex (male)	0.159	0.037	−0.028	0.009	0.004	<0.001
Birth weight (kg)	0.167	0.040	0.0001	0.000	<0.001	NA
Weight-for-age z-score						
Birth weight (kg)	−4.877	0.758	0.001	0.0002	<0.001	NA
Length-for-age z-score						
GA (weeks)	−15.416	4.565	0.275	0.129	0.038	<0.001
Birth weight (kg)	−5.956	1.081	0.002	0.0003	<0.001	NA
BMI-for-age z-score						
Birth weight (kg)	−2.213	0.907	0.001	0.0003	0.023	NA
Head circumference-for-age z-score						
Birth weight (kg)	−2.786	1.107	0.001	0.0003	0.003	NA

Data are parameter estimate ± standard error of measurement (SE) after accounting for infant birth weight. BMI—body mass index; GA—gestational age.

**Table A3:** Associations between infant and maternal body composition at 3 months postpartum.

Maternal Predictor	Intercept	SE	Slope	SE	Predictor	Infant Birth Weight
					*p*-Value	*p*-Value
Infant weight (kg)						
Weight (kg)	2.489	0.655	−0.013	0.006	0.025	<0.001
BMI (kg/m^2^)	2.836	0.646	−0.049	0.015	0.002	<0.001
FM (kg)	2.273	0.634	−0.022	0.009	0.014	<0.001
FM (%)	3.034	0.748	−0.029	0.013	0.026	<0.001
FFMI (kg/m^2^)	2.938	0.679	−0.083	0.029	0.006	<0.001
FMI (kg/m^2^)	2.408	0.628	−0.066	0.023	0.006	<0.001
Infant body mass index (kg/m^2^)						
Weight (kg)	14.308	1.327	−0.038	0.011	0.001	<0.001
BMI (kg/m^2^)	14.808	1.372	−0.108	0.032	0.001	0.001
FFM (kg)	14.535	1.366	−0.066	0.020	0.002	0.001
FM (kg)	13.621	1.304	−0.058	0.018	0.002	0.002
FM (%)	15.468	1.573	−0.072	0.027	0.010	0.011
FFMI (kg/m^2^)	15.160	1.423	−0.198	0.060	0.002	0.001
FMI (kg/m^2^)	13.876	1.328	−0.150	0.048	0.003	0.004
Infant head circumference (cm)						
FFM (kg)	37.037	1.396	−0.044	0.019	0.023	<0.001
FFMI (kg/m^2^)	37.791	1.431	−0.152	0.054	0.007	<0.001
Infant fat-free mass (kg)						
BMI (kg/m^2^)	2.549	0.456	−0.029	0.011	0.009	<0.001
FM (kg)	2.197	0.438	−0.013	0.006	0.032	<0.001
FM (%)	2.743	0.521	−0.020	0.009	0.030	<0.001
FFMI (kg/m^2^)	2.646	0.510	−0.051	0.024	0.042	<0.001
FMI (kg/m^2^)	2.293	0.435	−0.042	0.016	0.012	<0.001
FM/FFM	2.456	0.468	−0.715	0.337	0.039	<0.001
Infant fat-free mass index (kg/m^2^)						
Weight (kg)	11.053	1.161	−0.025	0.010	0.015	<0.001
BMI (kg/m^2^)	11.613	1.174	−0.084	0.028	0.004	<0.001
FM (kg)	10.634	1.116	−0.043	0.015	0.007	<0.001
FM (%)	12.242	1.343	−0.061	0.024	0.013	<0.001
FFMI (kg/m^2^)	11.849	1.327	−0.141	0.063	0.030	<0.001
FMI (kg/m^2^)	10.885	1.118	−0.122	0.041	0.005	<0.001
FM/FFM	11.372	1.210	−2.113	0.871	0.019	<0.001
Infant fat mass (kg)						
Weight (kg)	0.073	0.263	−0.005	0.002	0.041	<0.001
BMI (kg/m^2^)	0.194	0.265	−0.017	0.006	0.010	<0.001
FFMI (kg/m^2^)	0.337	0.288	−0.037	0.014	0.009	<0.001
FMI (kg/m^2^)	0.030	0.258	−0.020	0.010	0.038	<0.001
Infant fat mass index (kg/m^2^)						
Weight (kg)	1.409	0.768	−0.015	0.007	0.023	<0.001
BMI (kg/m^2^)	1.720	0.785	−0.049	0.018	0.010	<0.001
FFM (kg)	1.564	0.810	−0.027	0.013	0.047	<0.001
FM (kg)	1.119	0.757	−0.022	0.010	0.042	<0.001
FFMI (kg/m^2^)	2.148	0.851	−0.110	0.041	0.009	<0.001
FMI (kg/m^2^)	1.235	0.763	−0.059	0.028	0.041	<0.001
Infant fat mass (%)						
Age (years)	19.908	2.888	−0.119	0.056	0.039	<0.001
Infant fat mass to fat-free mass ratio						
Age (years)	0.236	0.052	−0.002	0.001	0.0495	<0.001
Weight-for-length z-score						
Weight (kg)	0.083	0.961	−0.028	0.008	0.001	0.069
BMI (kg/m^2^)	0.310	1.020	−0.069	0.024	0.005	0.15
FFM (kg)	0.264	0.985	−0.049	0.015	0.002	0.070
FM (kg)	−0.431	0.953	−0.041	0.013	0.002	0.15
FM (%)	0.738	1.159	−0.046	0.020	0.023	0.40
FFMI (kg/m^2^)	0.524	1.059	−0.126	0.045	0.007	0.15
FMI (kg/m^2^)	−0.285	0.983	−0.098	0.036	0.008	0.25
Weight-for-age z-score						
Weight (kg)	−4.451	0.739	−0.016	0.006	0.015	<0.001
BMI (kg/m^2^)	−4.128	0.743	−0.053	0.017	0.004	<0.001
FM (kg)	−4.728	0.721	−0.025	0.010	0.014	<0.001
FM (%)	−3.969	0.861	−0.030	0.015	0.048	<0.001
FFMI (kg/m^2^)	−4.044	0.783	−0.087	0.033	0.011	<0.001
FMI (kg/m^2^)	−4.594	0.722	−0.070	0.026	0.010	<0.001
Length-for-age z-score						
Age (year)	−3.828	1.382	−0.063	0.027	0.024	<0.001
BMI-for-age z-score						
Weight (kg)	−1.744	0.894	−0.017	0.008	0.025	0.003
BMI (kg/m^2^)	−1.518	0.923	−0.049	0.021	0.027	0.004
FFM (kg)	−1.588	0.905	−0.033	0.014	0.020	0.003
FM (kg)	−2.058	0.877	−0.026	0.012	0.032	0.005
FM (%)	−1.134	1.031	−0.035	0.018	0.0495	0.012
FFMI (kg/m^2^)	−1.284	0.946	−0.098	0.040	0.019	0.003
FMI (kg/m^2^)	−1.942	0.887	−0.068	0.032	0.041	0.007
Head circumference-for-age z-score						
FFM (kg)	−2.112	1.093	−0.034	0.015	0.023	<0.001
FFMI (kg/m^2^)	−1.733	1.152	−0.099	0.043	0.026	<0.001

Data are parameter estimate ± standard error of measurement (SE) after accounting for infant birth weight. BMI—body mass index; FFM—fat-free mass; FFMI—fat-free mass index; FM—fat mass; FMI—fat mass index; FM/FFM—fat mass to fat-free mass ratio.
